# Best Practices for Medication Utilization Evaluations in Postsurgical Pain Management

**DOI:** 10.1007/s40138-016-0121-2

**Published:** 2016-12-10

**Authors:** Brian Faley, John Fanikos

**Affiliations:** 1 Department of Pharmacy, Hackensack University Medical Center, 30 Prospect Ave., Hackensack, NJ 07601 USA; 2 Pacira Pharmaceuticals Medical Affairs, Division 5 Sylvan Way Parsippany, Hackensack, NJ 07054 USA; 3 Department of Pharmacy Services, Brigham and Women’s Hospital, 75 Francis Street, Boston, MA 02115 USA

**Keywords:** Drug use evaluation, Medication use evaluation, Postsurgical pain, Surgery

## Abstract

**Purpose:**

The purpose of this review is to provide guidance that aids in the practical design, implementation, and analysis of medication use evaluations (MUEs) for postsurgical pain management.

**Summary:**

Clinicians have long employed drug use evaluations or drug utilization reviews to ensure the safe and appropriate use of medications in a hospital, medical practice, or other healthcare setting. Although these approaches are valuable, there is a growing trend toward replacing these methods with the MUE, a performance improvement tool that focuses on assessing and improving medication use processes or medication treatment response with the goal of optimizing patient outcomes. Utilizing MUEs to assess patient outcomes and quality of life can be challenging in certain therapeutic areas such as pain management, where measurements of pain can be quantitative but are inherently subjective. Currently, there is little guidance on the development of MUEs that balance subjective and objective outcomes.

**Conclusion:**

MUEs continue to become the standard for quality improvement for optimizing care and ensuring optimal outcomes. This review of the literature provides guidance in post-surgical pain management, an area that requires measurement of both subjective and objective outcomes.

## Introduction

As the number of options for managing postsurgical pain continues to expand, healthcare providers and administrators have a growing need for a systematic method of evaluating and selecting which drugs or delivery systems are most likely to help their patients. Clinicians have long turned to drug use evaluations (DUEs) or drug utilization reviews (DURs) to ensure the safe and appropriate use of medications (e.g., dose and duration of treatment or avoiding drug/drug interactions), but those methods typically do not provide sufficient data to make an informed decision on whether drug A is better than drug B for managing pain in a specific postsurgical patient population.

To address those types of questions, the traditional DUE/DUR has been expanded into a medication use evaluation (MUE), a performance improvement tool that focuses on assessment and improvement of medication use processes or medication treatment response with the goal of optimizing patient outcomes [1, 2]. A comprehensive review of MUEs published in 2014 by Fanikos and colleagues recommended using MUEs in three following situations: when the benefit of the medication is unknown, when there are little data available to influence a choice between two medications, and when there is a need to analyze the process of medication prescribing, preparation, dispensing, administration, and monitoring [2]. MUEs, which have many parallels to investigator-initiated clinical trials, can be used to investigate individual drugs, therapeutic classes, disease states, or medication use processes [1]. In postsurgical pain, MUEs typically compare specific drugs, classes of drugs (e.g., opioids), or methods of drug delivery (e.g., local, oral, IV) [2].

A key aspect of MUEs is their emphasis on patient outcomes and quality of life [1–3]. This defining feature also provides the greatest challenge, as these types of endpoints often can be difficult to assess quantitatively and/or objectively [2]. The choice of endpoints can be particularly complex in pain management, as measurements of pain can be quantitative but are inherently subjective. It is possible to use more objective outcomes, such as functional improvement or length of hospital stay, but the relationship of those outcomes to postsurgical pain management frequently is unclear.

These challenges aside, the authors believe the value of MUEs in postsurgical pain management outweighs any potential hurdles and involved in creating a scientifically robust research protocol with carefully chosen endpoints. The purpose of this manuscript is to provide an educational resource to aid in the practical design, implementation, and analysis of MUEs for postsurgical pain. For ease of discussion, we have separated the process into five following sections: establishing responsibility, developing scope and objectives, designing the MUE, finalizing the protocol and analyzing data, and interpreting the results and making recommendations.

## Establishing Responsibility

Even MUEs that are meticulously designed and flawlessly executed are likely to be unsuccessful without engagement and support at several levels of the institution [2]. Most institutions’ task existing committees (e.g., Pharmacy and Therapeutics, Drug and Therapeutics, Quality Management) with overseeing the MUE process, including choosing the topics to be evaluated, establishing the policies and procedures to be followed, and implementing any necessary changes to prescribing or clinical practice based on the findings [1, 3].

Specific MUEs are typically managed by a collaborative, interdisciplinary team that includes a pharmacist, prescriber, nurse, administrator, and a representative of any other healthcare profession relevant to the MUE (e.g., lab technician, physical therapist, nutritionist) [1, 2]. For postsurgical pain, an MUE committee might consist of a pharmacist, a surgeon, an anesthesiologist, a nurse in the postanesthesia care unit, a floor nurse, and a physical therapist. MUEs are commonly spearheaded by pharmacists, but the roles of all team members may vary based on the goals and objectives of the MUE, the practice setting, and the available resources [1].

### Developing Scope and Objectives

Historically, the most common driver for the development and implementation of DURs, DUEs, and MUEs has been cost. However, the key benefit of MUEs is that they also can be effectively employed to address a wide range of issues related to patient-specific outcomes, such as safety, medication effectiveness, appropriate dosing, and quality standards [1–3]. Start the MUE process by clearly identifying the medical need or question in as much detail as possible [3]. For example, the statement, “We need to find out why we are using so much of drug A,” is insufficiently descriptive. Add specificity by considering questions such as: “Which medical specialty is prescribing drug A the most?”, “Has there been a sudden increase in use of drug A?”, “Is there concern about a specific adverse event with drug A?”, and “Are insurance companies refusing to cover drug A?” Depending on the answers, a more suitable statement of medical need might be, “In the last year, use of drug A increased 27% in the intensive care unit (ICU). However, drug A has been associated with higher rates of rash in acutely ill patients. “Is drug A being used appropriately in this setting?”

The next step is to translate the medical need into one or more specific, measureable objectives. Using the example above, consider how to define appropriate use. Is the concern over any use of drug A in the ICU incorrect dosing, use of drug A in combination with drug B costly, or some other issue? Depending on the responses, potential MUE objectives might be the following: [1] establish whether the increase in use is the result of a greater number of patients requiring drug A, or higher doses of drug A being prescribed, [2] determine the percentage of ICU patients using drug A who have subsequent new prescriptions for antihistamines or oral steroids to treat rash, and [3] compare the ICU length of stay between patients not exposed to drug A, patients exposed to drug A who did not develop a rash requiring additional medications, and patients exposed drug A who had subsequent new prescriptions for antihistamines or oral steroids.

Some institutions routinely forgo the systematic generation of MUE objectives, often because they feel the answers are obvious. However, these early decisions are critical to the design of the MUE, and devoting resources to developing a robust scope and measurable objectives is likely to save time and resources later in the MUE process.

#### Designing the Study

Though rarely as complex as a clinical trial, a well-designed MUE should follow a prespecified protocol that includes many of the same following elements: study design, population, endpoints, data collection, and statistical analyses.

##### Study Design

MUEs are typically performed at a single center, but they can be prospective (when the prescription is filled), concurrent (during treatment), or retrospective (after an endpoint has been reached or treatment is completed) [4]. When evaluating postsurgical pain management, MUEs are typically retrospective and compare two or more drugs/drug classes, delivery methods, or surgical techniques. The structure of the comparison (e.g., intervention vs. standard of care, matched case-control) depends on the objectives of the MUE and the available data. For example, an MUE by Kampe et al. compared outcomes from patients undergoing thoracotomy who were given one of two drugs, whereas a different MUE from Kelly and colleagues used a case-control design to evaluate the use of intravenous (IV) acetaminophen in knee arthroplasty [5, 6].

##### Population

When determining the population to be studied, many variables need to be considered, including sample size, time period for data collection, and inclusion/exclusion criteria. In most cases, the sample size and time period for data collection are based on practical rather than statistical considerations. There are no firm rules, but MUEs often include a few dozen to a few hundred patients who are treated within a 1- to 3-year period [5–8]. Larger sample sizes are generally desirable, but there is almost always a need to balance rigor and practicality. On one hand, extending the time period to include additional patients might introduce bias if any changes were made to the standard of care from the beginning to the end of data collection, but on the other hand, a change to the standard of care may present an opportunity for evaluation. For example, Kaplan et al. examined two following groups of patients undergoing total knee arthroplasty: a control cohort treated with standard of care between July 1, 2012, and December 31, 2012, and an intervention group treated with a new drug formulation between July 1, 2013 and January 31, 2014 [8].

Well-considered inclusion/exclusion criteria can strengthen an MUE by helping to eliminate bias introduced by interpatient heterogeneity. In postsurgical pain management, individual MUEs should be restricted to a single surgical intervention (e.g., total knee arthroplasty, thoracotomy, Roux-en-Y-gastric bypass) and, with the exception of the variable(s) under study, with as little variation as possible between surgeons, surgical procedures, and drug delivery techniques. Exclusion criteria can also be used to protect patient safety (e.g., by excluding patients with known hypersensitivity to opioids, bupivacaine, etc.) and preserve the integrity of the data (e.g., by excluding chronic opioid users or patients with incomplete documentation) [5–8].

##### Endpoints

The choice of endpoints is based on the objectives of the MUE. Ideal endpoints are objective (vs. subjective), measurable (either qualitative or quantitative), relevant, and use data that is reasonably easy to obtain [2]. When designing an MUE, it may be helpful to distinguish between variables (e.g., opioid dose, pain score) and the application of those variables to yield endpoints (e.g., difference in opioid dose, change in pain score) [2]. Table 1 [5–8] lists variables that are commonly used in MUEs for postsurgical pain, detailing the pros and cons of each and providing examples of potential endpoints.

**Table 1:** Potential endpoints for medication use evaluations in postsurgical pain [5–8]

Variable	Pros	Cons	Potential endpoints	Clarifying questions
Opioid use	• Objective• Easy to measure with prescription records	• Usage may vary widely between patients for subjective reasons (difference in pain threshold, AEs, tolerance)	• Change in opioid use over time via pill counts • Difference in total opioid use between treatment groups • Reduction in total morphine equivalents • Percentage of patients discharged on opioids • Constipation rates	• Are the opioids self-administered, dispensed on request, or administered when a predetermined threshold pain score is reached? • When does the measurement period begin/end? • What is a clinically meaningful difference?
Pain scores	• Easy to administer• Can be used with children and patients with low literacy	• Subjective• Likely to vary widely between patients	• Absolute change in pain intensity or pain relief over time • Percentage change in pain intensity/relief over time • Difference in pain scores between treatment groups • 30 and 50% responder analysis • Average pain score at discharge	• When does the measurement period begin/end? • What is the interval for data collection and the allowable variation (e.g., 6 ± 1 h, 24 ± 4 h)? • Are data collected when the patient is at rest or during activity (e.g., coughing after thoracotomy)? • What is a clinically meaningful difference?
Functional measures^a^	• Objective • Focused on patient outcomes • May be measured as part of standard care	• Changes may or may not be driven by pain	• Change in range of motion over time • Time to ambulation • Percentage of physical therapy sessions completed • Time to return to work/normal activities after surgery	• What changes in functional measures are appropriate to measure for a patient 24 h postsurgery? • Are the outcome and the use of pain medications temporally related (e.g., patients may taper off opioids long before they return to work)? • When are data collected? • What is a clinically meaningful difference?
Adverse events	• May be objective • May be captured as part of normal care, particularly if serious	• May be subjective • May or may not be associated with use of pain medication (e.g., constipation, infection)	• Incidence in each treatment group • Time to resolution • Percentage of patients with serious AEs in each treatment group	• Which AEs are of interest? • When does the measurement period begin/end? • What is a clinically meaningful difference?
Length of stay	• Objective• Should be easily accessible with computerized records	• May be affected by non–pain-related factors (e.g., comorbidities that slow healing, clinicians’ schedules, patient transportation)	• Difference of total length of stay between treatment groups • Differences in level of care (e.g., intensive care stay) • Percentage of patients exceeding a predetermined length-of-stay threshold (e.g., 3 days)	• When does the measurement period begin/end? • Does the precision of the endpoint match the precision of the data (e.g., reporting the mean length of stay in minutes when the data are routinely rounded to the nearest hour)? • What is a clinically meaningful difference?
Cost	• Objective• Should be easily accessible with computerized records	• Not a patient-oriented endpoint, which is the primary goal of an MUE (cost can be a component of an MUE but should not be the focus)	• Difference between treatment groups	• Does the cost include the drug only? Are there relevant differences in equipment, personnel time, etc., between treatments that should be included? • What is the cost basis (e.g., what institution bills vs. what insurance company or patients pay)? • What is a meaningful difference? • What is an appropriate tradeoff between cost and outcomes?
Patient satisfaction	• Focused on patient • Likely to encompass efficacy and safety	• Subjective • May be difficult to separate satisfaction with pain medications from other factors	• Average satisfaction score • Difference between treatment groups	• Is the endpoint general patient satisfaction with medical care, or should it be more focused on pain management? • What is the measurement period or interval? • What is a clinically meaningful difference?

*AEs* adverse events

^b^Choice depends on type of surgery; examples include pulmonary function, time to resume work/normal activities, physical therapy goals, range of motion, etc.

The table also includes a list of questions that may help define the endpoint more precisely and improve the quality and specificity of the data. One of the most critical questions to ask is, what is the clinical relevance of an endpoint? For instance, what is a meaningful difference in pain score or length of hospital stay in terms of patient outcomes? Would an endpoint of no overall change in pain score be clinically acceptable if it were accompanied by a significant increase in one or more functional measures? Unfortunately, there are too many potential confounders to suggest any firm rules, so decisions about what constitutes a meaningful difference should be made during the MUE design phase using the clinical knowledge and experience of the MUE team members [4].

Choosing endpoints in postsurgical pain management can be particularly challenging because of the inherently subjective nature of pain and the frequent interaction between the endpoints. For example, what does it mean if drug A improves a functional outcome (e.g., range of motion) without significantly lowering mean pain score in comparison with drug B? Does that result imply that drug A is unable to effectively lessen pain, or does it suggest that patients taking drug A can tolerate higher levels of activity without increasing pain than patients taking drug B? The MUE design phase is an ideal time to consider these types questions and, if possible, identify some method of extracting that information during the study.

##### Data Collection Plan

This part of the MUE study design should detail who, what, where, when, and how the data will be collected. Examples for each category are provided in Table 2 [2, 5–8]. Some endpoints, such as pain scores or satisfaction ratings, may require data from the patients themselves. Fanikos and colleagues encourage MUE designers to prospectively consider whether the collection procedures have the potential to significantly disrupt or negatively impact patient care, and to adjust the study design accordingly [2].

**Table 2:** Examples of data collection procedures [2, 5–8]

Who	• MUE team members • Additional point-of-care providers • Other designees (e.g., medical students and interns) with proper training and supervision
What	• Demographics (e.g., age, gender, weight) • Designated primary endpoint(s)—subjective/objective • Secondary endpoint(s) —subjective/objective • Potentially confounding factors (e.g., comorbidities, simultaneous surgeries, opioid tolerance)
Where	• At point of care • Retrospectively extracted from a database (e.g., medical or prescription records, insurance claims)
When	• Prespecified time period (e.g., from January 2014 to December 2014) • At what interval (e.g., every 6 h, once per wk)
How	• Collated on paper or in a spreadsheet created by the person collecting the data • Using paper or electronic data collection forms created by the MUE team • Using validated surveys or scores (e.g., pain, patient satisfaction) • Reports and queries from databases

*MUE* medication utilization evaluation

##### Statistical Analysis

One of the biggest challenges faced when designing an MUE is the ability to identify and resolve the numerous statistical subtleties that can significantly impact outcomes. Just like any clinical study, the identification of appropriate endpoints, establishing clinically relevant controls, and establishing the appropriate statistical test for study objectives are keys when developing the statistical plan for an MUE. There are several statistical questions that the investigators should consider as a part of the statistical plan (Table 3) [5–8]. When possible, consultation with an experienced statistician may be helpful in ensuring a robust design.

**Table 3:** Key statistical questions to address when designing a medication use evaluation [5–8]

Design	• Does the study have appropriate controls or comparator groups? • Are all the needed analyses specified in the study design? • How will incomplete or missing data be addressed?
Choice of statistical test	• Will the data be checked for normal distribution before applying descriptive statistics (e.g., mean, standard deviation, median)? • What are the criteria for including or excluding outliers? • What is the appropriate comparison test for the anticipated data (e.g., *t* test, Mann-Whitney U test, chi-square test)?
Choice of statistical model	• If needed, what is the appropriate statistical model for the anticipated data (e.g., linear regression, logistic regression, analysis of variance)? • How will the model be adjusted for potentially confounding factors (e.g., unequal baseline characteristics, multiple surgeons, different surgical approaches or techniques)?
Choice of significance threshold	• What is the threshold for significance? An explanation may be required if anything besides *p* < 0.05 is chosen. • How will results that are statistically significant but not clinically different be interpreted?

##### Finalizing the Protocol and Analyzing Data

MUEs almost always involve protected health information, so once the design is complete, the next step is to obtain approval from an institutional review board. Training on the protocol should be provided to relevant staff, and periodic quality checks are highly recommended throughout the data collection period [2]. Once the database is complete, proceed through the statistical analysis exactly as planned. It is inappropriate to add or modify endpoints after the data are collected, unless the results are clearly labeled as a post-hoc analysis and interpreted with caution.

##### Interpreting Results and Making Recommendations

When the data have been analyzed, the MUE team should meet and discuss the interpretation of the results from both a statistical and a clinical perspective. It may be worthwhile to reexamine some of the questions and issues considered during the design phase. Did the MUE meet its objectives? Was the appropriate patient population included? Were there any known shifts in the standard of care during the data collection period? How confident is the team in the quality and accuracy of the data? Do the results show a clinically relevant difference between drugs? What are the possible explanations for the outcomes? When possible, maintain the spirit of an MUE by prioritizing the results of patient-focused endpoints [2]. Also reconsider the tradeoffs between the medications under study. For example, is it worthwhile to spend more on a drug A if does not reduce mean pain scores but does significantly improve functional status (e.g., range of motion) compared with drug B?

The end product of the discussion should be a consensus as to how the MUE results should be applied. Is a change in prescribing or standard of care warranted? If yes, who needs to be notified? What is the appropriate time period for implementation of the change? What is the best way to communicate the information? Is there a need for a follow-up MUE to assess compliance to the change or success of its implementation? This meeting is also an ideal time to assess the MUE process itself and plan for any needed improvements [3, 4]. Since literature in this area is scant, also consider publishing a detailed account of the MUE design, outcomes, and data interpretation.

##### Case Example: Postsurgical Pain Management Medication Use Evaluations

To elucidate some of the intricacies related to the development of an MUE in the area of postsurgical pain management, we present the following case.

A clinical pharmacist with responsibilities in the surgical unit has received several requests from her anesthesiologist and surgical colleagues regarding the use of a newly approved medication for the management of surgical pain. The new medication (referred to for the purposes of this example as doloremol) has provisional status from the Pharmacy and Therapeutics committee (P&T) and requires adherence to a strict protocol for its use. Doloremol is a non-opioid, centrally-acting analgesic that can be delivered intravenously during surgery, and there is recently published data supporting its use in orthopedic surgery. While many of the surgeons at this institution have found doloremol to be beneficial for their patients, there are no published studies on the medication’s effect on outcome measures, such as reduction of opioid use or impact on length of stay. The clinical pharmacist determines that a MUE should be conducted to determine if use of doloremol should become a permanent part of the postsurgical management pain management protocol for her unit.

##### Establishing Responsibility

Because the surgery and anesthesiology departments were already interested in providing doloremol for patients, the clinical pharmacist identified individuals in these departments with whom to collaborate for the development of the MUE protocol and obtained preliminary authorizations from these departments.

##### Developing Scope and Objectives

Prior to determining any elements of the MUE with her assembled team, the pharmacist evaluated the literature for additional information on doloremol that might inform MUE development or serve as background information on the unmet need for a treatment like doloremol. The pharmacist searched the following databases: PubMed (http://www.ncbi.nlm.nih.gov/pubmed), the Cochrane Database of Systematic Reviews (http://www.cochranelibrary.com/cochrane-database-of-systematic-reviews/index.html), the National Health Service Economic Evaluation Database and Database of Abstracts of Reviews of Effects (http://www.crd.york.ac.uk/crdweb/), and the Central Register of Controlled Trials (http://www.cochranelibrary.com/about/central-landing-page.html). Her search identified additional doloremol dosing approaches that were used by several other institutions to manage several surgical conditions. One of these dosing regimens was added to the MUE analysis as a comparator to the dosing regimen currently in place in the surgical unit.

The MUE developers determined that the objective of this study would be to evaluate the effect of IV doloremol on pain, opioid use, and hospital length of stay when used in orthopedic surgery. The hypothesis was that, when administered during hip replacement surgery, IV doloremol (administered via either dosing arm) compared to a historical control would reduce the morphine equivalent dose of postsurgical opioids, with no difference in pain scores, reduced length of stay, and/or increased physical therapy time within the first 48 h after surgery.

##### Designing the Study

While approaches to designing MUEs vary from institution to institution, the MUE described in this scenario required a series of forms to be completed, to outline goals and objectives, the need for the study, inclusion and exclusion criteria, study procedures, statistical analyses, anticipated safety risks to the patients, and procedures for ensuring confidentiality. In addition to completing forms pertaining to study details, the team also needed to fill out forms for data gathering, consent, and storage and distribution of the medication. Many of these forms were standardized by the institution for use with research activities and may be available at other facilities. It is important to obtain these forms early in the process to facilitate MUE protocol development. If a team is developing new forms, such as those for data collection, it is important to trial these forms for a few test patients prior to finalizing, which will allow the team to identify potential barriers and provide solutions prior to implementation.

In order to assess the primary endpoint, reduced morphine equivalent dose of postsurgical opioids at 48 h postsurgery, the clinical pharmacist had to develop an assessment procedure to ensure that each patient’s pain and opioid use were assessed immediately with postsurgery 48 h later. In this case, the clinical pharmacist had access to patients’ opioid medication doses and administration timing through the electronic medical record (EMR). The secondary endpoints, including difference in pain scores, reduced length of stay, reduced costs, and/or increased physical therapy time also would be readily available through the EMR. All adverse reactions and toxicities would be recorded in the subject’s EMR as well.

The pharmacist and her colleagues established that differences in means would be considered significant for *p* < 0.05. A one-tailed *t* test was selected to show decreased opioid use with IV doloremol, and two-tailed *t* tests were intended to show no difference with respect to mean change in absolute pain score and mean cost endpoints.

It was determined that 50 prospective patients would be necessary for each dosing arm to conduct the statistical analyses. Additionally, there would be a need to evaluate 200 historical controls, which would include patients treated in the surgical unit over a 4-year period.

##### Finalizing the Protocol and Analyzing Data

The proposal forms were completed by the clinical pharmacist and were given to the rest of the MUE team for review prior to submission. Depending on the institution, proposals usually will need to be submitted to the institutional review board (IRB) and potentially other internal peer-review committees to allow for objective oversight to ensure patient’s safety. Since the pharmacist’s institution is an academic one, the MUE proposal also was submitted to the university IRB. In some cases, these submissions can be done in parallel.

The P&T committee and the IRB group reviewed the surgical unit protocol and provided direction to refine several aspects of the proposal. Once refined and approved, the MUE team took 2 weeks to educate the various departments that would be exposed to the study protocols, including pharmacy, surgery, anesthesiology, nursing, physical therapy, and administration.

##### Interpreting the Results and Making Recommendations

The study was conducted over a 6-month period and evaluated 100 patients undergoing hip replacement surgery using either of the two dosing regimens of doloremol, along with review of data from 200 historic controls. The data collected from the MUE was then compiled and analyzed by the MUE working group. A summary of the data was presented to the P&T committee so its members could assess the potential benefit of changing the surgical unit protocol for perisurgical pain management. In other facilities, this data should be shared with all of the departments taking part in the MUE, because elements of the data may lead to improvements in processes within other departments or serve as learnings for future MUEs.

In addition to sharing the data with internal stakeholders, it is important to present the data externally. Surgical, anesthesiology, and pharmacy congresses would all be interested in learning about the development and value of an MUE like the one described in this case. It is important to involve authors who helped to develop the MUE protocol and are relevant to these audiences (e.g., having a surgeon on the MUE team serve as lead author for a surgical congress). Additionally, it is important to publish this information in a peer-reviewed publication. Journal article authorship should be reserved for individuals who were involved with the design and execution of the MUE.

##### Future Directions

At present, MUEs typically are performed at a single institution or within an integrated healthcare system. The next step in the evolution of MUEs is the development of multicenter protocols, which, in transcending the current practice of extracting data from claims databases or electronic medical records from a single site, would increase sample sizes and allow for evaluation that takes into account variations in geographical location, type or size of institution, etc. Anyone designing and implementing a multicenter MUE is likely to encounter several challenges, including coordinating a single protocol among several investigators, obtaining approval from multiple IRBs or ethics committees, ensuring universal data collection and quality control procedures, and securing funding for staff time at each site. However, multicenter MUEs would offer unique opportunities to decide which data to collect and to include endpoints, such as pain scores, functional outcomes, or patient satisfaction, that may not be captured in any single database.

## Conclusions

MUEs can be a valuable tool in the effort to improve patient outcomes in postsurgical pain management. Typically designed and conducted by an interdisciplinary team (e.g., pharmacists, prescribers, nurses, administrators) operating with the consent and support of the organization’s leadership, the most effective MUEs have specific objectives and a scientifically robust study design and data analysis plan. Results should be interpreted with a focus on clinical relevance and improved patient outcomes, and any recommended changes in prescribing behavior or standard of care that result should be clearly communicated to all stakeholders. A system to monitor compliance and incorporate feedback on the MUE process is also recommended.
